# Depression among Asian Americans: Review and Recommendations

**DOI:** 10.1155/2011/320902

**Published:** 2011-09-27

**Authors:** Zornitsa Kalibatseva, Frederick T. L. Leong

**Affiliations:** Michigan State University, East Lansing, MI, USA

## Abstract

This article presents a review of the prevalence and manifestation of depression among Asian Americans and discusses some of the existing issues in the assessment and diagnosis of depression among Asian Americans. The authors point out the diversity and increasing numbers of Asian Americans and the need to provide better mental health services for this population. While the prevalence of depression among Asian Americans is lower than that among other ethnic/racial groups, Asian Americans receive treatment for depression less often and its quality is less adequate. In addition, the previous belief that Asians somatize depression may become obsolete as more evidence appears to support that Westerners may “psychologize” depression. The cultural validity of the current DSM-IV conceptualization of depression is questioned. In the course of the review, the theme of complexity emerges: the heterogeneity of ethnic Asian American groups, the multidimensionality of depression, and the intersectionality of multiple factors among depressed Asian Americans.

## 1. Introduction

The goal of this paper is to provide a review of the prevalence, manifestation, assessment, and diagnosis of depression among Asian Americans. Before the authors discuss these issues, they examine the current demographics of Asian Americans. The review reveals the complexity of depression among Asian Americans, as the disorder seems to be more multifaceted, and the population is more heterogeneous compared to previous conceptualizations of depression among Asian Americans in research and clinical practice. Finally, the authors provide recommendations for future research and practice by emphasizing the heterogeneity of Asian Americans, the multidimensionality of depression, and the intersectionality of various factors that may affect the experience of depression. The current review aims at contributing to the existing literature in several ways. First, there is a dearth of reviews of depression among Asian Americans, and the conducted empirical studies need to be reviewed using existing theoretical frameworks. Second, previous reviews concentrated on Asian American elderly only (e.g., [1]) or provided an extensive list of studies that have been conducted with different age groups (e.g., [2]). Therefore, this review differs by focusing on the manifestation, assessment, and diagnosis of depression among Asian Americans. In addition, the overarching theme of this review is the complexity and multidimensionality of depression among Asian Americans. Lastly, this review not only attempts to combine theory and empirical findings, but also provides recommendations for future research and practice.

## 2. Demographics of Asian Americans

Asian Americans are the fastest-growing minority group in the United States [3]. According to the 2010 Census, Asian Americans number 14,674,252 individuals (4.8% of the US population), which represents a significant increase of 43% compared to the 2000 Census when Asian Americans numbered 10,242,998 (3.6% of the US population). In terms of subgroups, Chinese Americans make up the largest Asian group in the United States at 0.9% of the country's population, followed by Filipino Americans (0.7%), Asian Indians (0.6%), Korean Americans (0.4%), and Japanese Americans (0.3%). For convenience, the US Census Bureau grouped Pacific Islanders together with Asian Americans, which resulted in a designation of Asian Pacific Islander Americans for this ethnic minority group. As of the 2000 Census, Pacific Islanders who are immigrants or descendents of immigrants from one of the Pacific Islands to the United States, including Hawaii, Samoa, Fiji, Guam, and the islands of Micronesia, were officially separated from the Asian Americans. 

As noted above, some Asian groups such as the East Asians (Chinese, Japanese, and Korean) and Indians are economically and politically more powerful than the Southeast Asians such as the Vietnamese, Hmong, Cambodians, and Laotians. For example, as pointed out by Li and Wang [4] “in the 2000 US Census, the poverty rate for Asians as a whole is only 10%. However, when Asian Americans are broken down by ethnicity, there is a wide range of poverty rates, varying from 6% for Filipinos to 38% for Hmong, with Japanese at 10%, Chinese at 14%, Vietnamese at 16%, and Cambodians at 29%. For example, according to Census 2000, only 22.85% of Vietnamese Americans hold a bachelor degree or higher, as compared to Korean at 49.2%, Japanese at 41.9%, Filipino at 42%, Chinese at 51.6%, and Asian Indian at 63.8%” [4, page 7].

According to Census 2000, the term “Asian” refers to people having origins in any of the original peoples of the Far East, Southeast Asia, or the Indian subcontinent (e.g., Cambodia, China, India, Japan, Korea, Malaysia, Pakistan, the Philippine Islands, Thailand, and Vietnam). Asian groups are not limited to nationalities, but include ethnic terms, as well. When referring to Asian Americans in this article, we include both immigrants from Asian countries to the US (first generation) and Americans of Asian descent (second, third, or fourth generation). Studies of Asian populations will also be discussed because they may be relevant to first generation Asian Americans. The comparison group that is most often used is European Americans and refers to people who originated from any of the peoples of Europe, the Middle East, or North America.

## 3. Prevalence of Depression among Asian Americans

According to the World Health Organization (WHO), major depression is reported as the leading cause of disability worldwide and the fourth largest contributor to the global burden of disease [5]. Major depression is a chief public health problem, and it is projected to be the second largest contributor to global disease burden by 2020 [6]. The lifetime prevalence rates of depression among European Americans according to the Diagnostic Statistical Manual (DSM-IV) range from 10% to 25% for women and from 5% to 12% for men [7]. Depression has been identified in all countries and among all ethnic and racial groups that have been studied [8–10]. The ubiquity and the serious consequences of major depression call for prompt actions in increasing our understanding of its etiology, phenomenology, assessment, diagnosis, and treatment. 

Among Asians and Asian Americans, reports about the lifetime prevalence of depression vary. The Chinese American Psychiatric Epidemiological Study (CAPES) indicated a relatively low lifetime prevalence rate of major depressive disorder (6.9%) among Chinese Americans [11]. Takeuchi et al. [12] reported that 9.1% of Asian Americans in the National Latino and Asian American Study (NLAAS) endorsed any affective disorder in comparison to 17.9% of non-Latino Whites, 13.5% of Hispanics, and 10.8% of non-Hispanic Blacks in the National Comorbidity Study-Replication (NCS-R; [13]). However, other studies suggested that the prevalence of depression among Asian Americans is equivalent or greater than that among European Americans (e.g., [14]). Chang [15] suggested that while Asian Americans reported significantly lower rates of depression than European Americans, their rates were overall higher than their overseas Asian counterparts.

More recently, Jackson et al. [16] have examined the prevalence rates of major depressive episode (MDE) among the various racial/ethnic groups in the Collaborative Psychiatric Epidemiological Surveys (CPES), which included the NLAAS. The NLAAS sampled three Asian ethnic groups: Filipinos, Vietnamese, and Chinese, and a fourth group included Other Asian. According to Jackson et al., the Asian ethnic groups reported the lowest rates of lifetime MDE compared to all others (non-Latino Whites, Hispanics, Caribbean blacks, and African Americans), and Filipinos were the ethnic group with the lowest rate (7.2%). In addition, Jackson et al. compared the prevalence rates of MDE for US born and non-US born participants and consistently found that among the Asian ethnic groups non-US born participants reported lower prevalence rates. Specifically, the MDE prevalence rate for US born Chinese Americans was 21.5% as opposed to 7.7% for the non-US born Chinese Americans. In conclusion, Jackson and colleagues emphasized the need to examine the interactions of culture, race, ethnicity, and immigration in future depression research of diverse populations. 

Kirmayer and Jarvis [17] provided a review of the cultural variations of prevalence, manifestation, mechanisms, and treatment of depression across different countries. Cross-national comparative community studies found that the prevalence of lifetime depression in Taiwan and Korea was 1.5% and 2.9%, respectively, as opposed to 5.2% in the United States [10]. The remarkably low prevalence rates in Taiwan and Korea may indicate differences in reporting of distress or possible protective effects of family and social support [17]. More recently, the International Consortium in Psychiatric Epidemiology examined data from 10 countries and found that Asian countries reported the lowest rates (3.0% in Japan) while Western countries reported the highest prevalence (16.9% in the United States; 15.7% in the Netherlands; [18]). One critique that Kirmayer and Jarvis made regarding the existing cross-national comparisons of prevalence rates was the existence of methodological issues in the studies. In particular, the instruments that were employed (e.g., the Composite International Diagnostic Interview or the Diagnostic Interview Schedule) use specific probes and assess symptoms that have been already identified in the United States as criteria for depression. However, variations in context and in symptomatology may result in biased estimates of depression prevalence [19]. 

Yang and WonPat-Borja [2] provided an overview of depression among Asian American youth, adults, and elderly. The authors concluded that although adult studies indicate high levels of depressive symptoms, the prevalence of MDD among Asian Americans in community samples is moderate to low. In addition, they suggested that recent Chinese immigrants and outpatient Asian American girls have been at higher risk for MDD than other groups [20, 21]. Overall, the prevalence rates of depression among Asian Americans are higher than those among Asians in Asia, and researchers have speculated that the difference may be due to acculturative stress. 

Regardless of the true prevalence of depression among Asian Americans, it has been established that once they have a mental disorder, it tends to be very persistent, and they are less likely to seek treatment for psychological problems than European Americans [22, 23]. Moreover, Alegría et al. [24] found that Asian Americans with a past-year depressive disorder were significantly less likely to access any depression treatment and to receive adequate care compared to non-Latino Whites. Thus, if Asian Americans suffer from depression, they may be less likely to have the disorder detected and treated, which may result in a worse prognosis [25].

## 4. Expression and Phenomenology of Depression among Asian Americans

The observed health disparities in depression treatment call for a closer examination of the manifestation and experience of depression among Asian Americans. The Western conceptualization of mental health relies on the notion of Cartesian dualism, considering the mind and the body as separate entities. The division of “psyche and soma” in Western medicine assumes that psychology and psychiatry deal with disorders of the mind and emotions while somatic medicine treats the body and its disorders [26]. However, this partition has proven to be quite controversial since all mental disorders according to the DSM [7] and ICD [27] classifications include somatic components. For instance, the current diagnosis of depression relies on both psychological and somatic symptoms. Interestingly, previous research implies that Westerners often describe depression in relation to concepts like guilt, individualism, decision-making, and self-control [28]. In addition, the affective aspect of depression has been suggested to receive more emphasis in North American samples than in Asian samples [29]. In contrast, Eastern experience of depression may reflect the integration of body and mind, which would explain the widespread occurrence of somatic symptoms in place of affective ones or the lack of differentiation between the two realms [30, 31].

The chief symptom in major depression in the West is considered to be sadness or depressed mood. However, in many societies people who suffer from major depression do not complain primarily of sadness. The symptoms that stand out for those people may be changes in appetite, headaches, backaches, stomachaches, insomnia, or fatigue [32]. Such symptoms and complaints would take people suffering from depression to their primary care doctor, and they may be less likely to be diagnosed with a mental disorder. 

One of the proposed explanations for the emphasis on somatic symptoms among Asian Americans has been the holistic representation of mind and body. Support for this proposition has been found in previous research on depressive symptoms among Asian Americans that examined the factor structure of the Center for Epidemiological Studies Depression Scale (CES-D). The CES-D assesses four domains of depression: negative/depressed affect, positive affect, interpersonal problems, and somatic symptoms [33]. However, these dimensions do not always hold, and fewer factors often emerge among ethnic/racial minority populations [34–36]. For example, Edman et al. found that in a sample of Filipino American adolescents, only two factors provided a reasonably good fit: the first one included depressed affect, somatic complaints, and interpersonal problems and the second one consisted of the positive affect items. This finding implies that depressive symptoms may cluster in a different way among Asian Americans. In addition, Kanazawa et al. [37] investigated cultural variations in depressive symptoms among Native Hawaiians, Japanese Americans, and European Americans using the CES-D and found that Japanese Americans reported lower levels of positive affect compared to European Americans. This discrepancy was attributed to the differences in emotion regulation rather than in levels of depression. Additionally, Lu et al. [38] examined the CES-D in a sample of Hong Kong Chinese and Anglo American students. While the authors found support for four factors in both samples, they observed a tendency among the Chinese participants to report somatic symptoms and a tendency among Anglo Americans to report both somatic and affective symptoms. Furthermore, Lu and colleagues [38] concluded that American participants considered somatic and affective experiences as two different dimensions that comprise depression equally, and Chinese individuals were more likely to report their somatic symptoms, as opposed to their depressed feelings despite their awareness of the psychological problem. The observed tendency among the Chinese participants to concentrate on somatic symptoms is arguably more socially acceptable and may be related to the assumption that a cure can be found more easily for such complaints [38].

Despite the various explanations that have been proposed to elucidate somatization, researchers recently have offered an alternative hypothesis [29]. A recent study by Ryder and colleagues has explored depressive symptom presentations among Chinese and Euro-Canadian outpatients and concluded that the type of assessment (spontaneous problem report, symptom self-report questionnaire, or structured clinical interview) influenced the type and frequency of the symptoms that the patient reported. In this study, Chinese outpatients were found to report more depressive somatic symptoms in spontaneous report and structured interviews while Euro-Canadian outpatients reported significantly more depressive affective symptoms (e.g., depressed mood, anhedonia, worthlessness, guilt) in all three assessment modalities. Based on their findings, Ryder and colleagues suggested that researchers may have spent too much time on discussing Chinese somatization of depression. Instead, they argued that it was more likely that Westerners overemphasized the affective or psychological aspects of depression compared to other cultures. This phenomenon is referred to as the “psychologization” of depression (Ryder et al.). 

In support of this argument, Yang and WonPat-Borja's [2] review of psychopathology among Asian Americans reiterated that among those who suffer from depression, somatic complaints go along with affective complaints as opposed to the previously held view that somatic complaints serve as a denial of affective symptoms. Interestingly, Chentsova-Dutton and colleagues have proposed the cultural norm hypothesis, which predicts that depression will decrease a person's ability to react in a culturally appropriate way to positive and negative emotions [39, 40]. In support of this proposition, the researchers found that depressed European Americans had difficulty expressing sadness openly when watching a sad movie, while East Asians showed increased emotional reactivity (i.e., cried more) compared to nondepressed participants. These findings provide evidence that depressed East Asians express sadness affectively and not only somatically, as previously claimed.

## 5. Assessment and Diagnosis of Depression among Asian Americans

One of the debates in the field of cultural psychopathology relates to the universality of normality and pathology [41, 42]. Researchers reviewed empirical evidence on whether psychiatric disorders are etic (culture-universal phenomena) or emic (culture-specific phenomena). To illustrate this point with the case of depression, an etic view would assume that all people express depression similarly and universal diagnostic criteria can be applied without cultural biases. Conversely, an emic perspective of depression would claim that even if universal depressive symptoms existed, there is cultural variability in their expression [43, 44]. Moreover, cultural settings may define what is considered abnormal, the amount and the nature of the symptoms that are required for impairment, the course of the disorder, and the most appropriate treatment. 

The DSM and ICD classification systems adopt a relatively etic or universal view of mental disorders because they minimize the role of culture in the diagnostic classification. At the same time, Fabrega [42] insisted that Western European psychiatric nosology may be ethnocentric because it reflects specific histories and cultures. The conceptualization of psychiatric illness and diagnosis indicates conventions about normality and abnormality of behavior, personhood, social behavior, and nature of disease that emerged in a society. According to Fabrega, the DSM-IV employed language categories and epistemologies of scientific objectivism, which suggests universality of psychiatric disorders. However, these diagnostic categories ignored the consideration of symbolic personal characteristics, such as motives, intentions, social standing, power, spiritualism, values, ethics, and life goals. The minimization of such personal characteristics automatically assumes emphasis on the conceptualization of personhood in Western societies, which accentuates autonomy, voluntarism, and individualism (Fabrega). Yet, there is evidence to suggest that other characteristics of the self may be more valued in Asian cultures, such as interdependence, relatedness, and collectivism [45, 46]. Therefore, clinicians need to be cognizant of, and attend to, to the inherent cultural biases in our current psychiatric nosology, especially when diagnosing depressive disorders. In addition, it is worth noting that some of the DSM-IV symptoms are directly related to Judeo-Christian religious concerns with guilt, sin, sloth, despair, and worthlessness [47]. However, these presentations may not be equally applicable among Asians who may embrace different religions and societal norms.

Marsella [48, 49] proposed that one of the main cultural influences of depressive experience and disorder is the concept of personhood held by a particular cultural tradition. On one end of the continuum, cultures are characterized by individuated self-structures, abstract languages, and a lexical mode of experiencing reality. In such cultures, individuals have “objective epistemic orientation,” express affective, existential, cognitive, and somatic symptoms, and experience an increased sense of isolation, detachment, and separation. In contrast, cultures at the other end of the continuum emphasize unindividuated self-structure, metaphorical language, and imagistic mode of experiencing reality. In these instances, individuals having “subjective epistemic orientation,” may express predominantly somatic symptoms and often experience depression in somatic and interpersonal domains encouraged by their society. To illustrate this, Western cultures value individuality and responsibility and often the associated depressive symptoms are related to the loss of personal control expressed in helplessness and powerlessness. On the other hand, in certain Eastern Asian (e.g., Chinese, Japanese, and Korean) societies, there is a strong emphasis on selfless subordination, and the loss of control does not have such a negative connotation, which may lead to different manifestation of depression, in which helplessness is not expressed as a symptom.

The assessment of depression among Asians and Asian Americans for research purposes has to be conducted using primarily self-report measures and structured or semistructured clinical interviews. Leong et al. [50] provided a review of the existing literature examining self-report measures of depression in East Asia. The authors noted that many researchers attempted to explain diverging patterns of depressive symptomatology based on results from Western instruments of depression. In particular, they criticized the lack of studies to test the reliability and validity of the Western questionnaires when used with Asians. While Leong et al. [50] concluded that the existing Western depression measures are adequate instruments for the assessment of depression, they also suggested that applying the current Western ethnocentric conceptualization of depression to other racial and ethnic groups and cultures result in missing expressions of depression that are culture specific and unique to Asians.

## 6. Recommendations

After reviewing the prevalence, manifestation, assessment, and diagnosis of depression among Asian Americans, we would like to provide recommendations for future research and clinical practice. The overarching theme of our recommendations is the complexity of this topic and the need to recognize it and understand it. In particular, we will concentrate on three areas that present the intricacy of understanding depression among Asian Americans: (1) the heterogeneity present between and within ethnic Asian groups and the associated sampling problems, (2) the multidimensionality of depression, and (3) the intersectionality, or complicated interactions between various factors, such as gender, race, ethnicity, immigration, acculturation, language proficiency, and socioeconomic status among depressed Asian Americans.

### 6.1. Heterogeneity and Sampling Problems with Asian Americans

As mentioned earlier, Asian Americans constitute a very heterogeneous, group and this presents a unique methodological challenge in conducting clinical research with this population. Due to the geographical distribution of Asian Americans in this country, it is quite challenging for researchers to find representative and adequate sample sizes when conducting research with Asian Americans. Outside of the major states of California and New York, obtaining samples of Asian Americans for clinical studies is akin to sampling rare events. Hence, it is quite difficult to satisfy the traditional research criteria of using random sampling techniques in order to achieve a representative sample that would allow for the greatest generalizability of the findings. As Sue et al. [51] have noted, the difficulty of this problem of sampling rare events and finding adequate sample sizes has caused some researchers to collapse ethnic categories among the races, which in turn creates methodological problems that limit the interpretation and generalizability of research findings. Sue et al. [51] further noted that this practice is common in studies of Asian and Pacific Islander Americans, which comprise of highly heterogeneous and diverse groups of people from Asia and the Pacific. Sue et al. [51, page 62] issued the following warning regarding improper sampling: “By considering the Asian and Pacific Islander Americans as a homogeneous group, we ignore sociohistorical, cultural, economic, and political diversity”. In addition to collapsing across heterogeneous groups, small subpopulation sizes also encourage researchers to resort to using convenience samples when conducting research with racial and ethnic minority groups [51]. Such samples suffer from the lack of representativeness, which in turn severely restricts the generalizability of the findings. 

Such overdependence on convenience samples can be problematic. For example, studies with convenience samples can significantly skew the results of meta-analyses, which attempt to examine the cumulative effects of our research in any particular domain. It may be useful for such meta-analytic studies to monitor, compute, and address the issue of convenience sampling in addition to the “file drawer” problem. Moreover, there has also been a pattern of many of these studies of depression to be conducted among college and university samples rather than community and clinical samples. 

Furthermore, there is also the problem of the politics of numbers such that the larger racial and ethnic minority groups have tended to receive most of the research attention. For example, in the Epidemiological Catchment Area (ECA) studies funded by the National Institute of Mental Health (NIMH), African Americans were oversampled but not Asian Americans. It was not until several decades later that the NIMH finally funded epidemiological studies, such as the National Latino Asian American Study (NLAAS). This pattern was probably also influenced by the stereotype of Asian Americans as the “model minority” who are viewed as highly successful in terms of educational achievement, economic attainment, and psychological adaptation in the United States (see [4]). 

Therefore, it is important for researchers conducting studies of depression among Asian Americans to be aware of this heterogeneity problem. Given the difficulty of obtaining adequate samples of Asian Americans, especially clinical samples, it is expected that studies will continue to consist of various subgroups (e.g., Chinese, Japanese, Filipino) combined together to provide adequate power for statistical analysis. However, it would be important to provide information about the various subgroups that have been combined in the study. A study consisting primarily of Vietnamese Americans will have different implications than one consisting primarily of Asian Indians. In addition, where possible, studies of specific Asian American subgroups (e.g., Chinese or Japanese Americans) should also be carried out when such samples can be obtained in significant numbers. Requiring only specific Asian subgroup studies to be published in journals would significantly hamper the field while not conducting specific subgroup studies, where possible, would also slow our progress. For now, it will require both the aggregated and disaggregated studies of depression among Asian Americans to help us advance the field.

Owing to the difficulties of obtaining adequate and representative samples for cross-cultural clinical research, it appears that the secondary analysis of archival dataset is increasing in frequency. Secondary analysis of archival clinical data offers an additional strategy for conducting studies of depression among Asian Americans. For example, the recent release of the NLAAS dataset to the scientific community for secondary analysis has resulted in a rise of clinical studies on Asian American mental health, including studies of depression. Perhaps related to this trend, the American Psychological Association has recently published a book on the method of secondary data analysis [52]. In addition, the value of this approach has also been recognized by various National Institute of Health (NIH) agencies, which have started funding secondary data analysis studies. Given the sampling problems discussed above, the use of secondary analysis of clinical data may well be an important alternative approach. If the NLAAS dataset is representative of this trend in secondary analysis, it would, therefore, be important for NIH to continue to fund these large national studies of racial and ethnic minority mental health, which can then be released to the scientific community for secondary analysis.

### 6.2. Depression as a Multidimensional Construct

One of the major problems with ethnocultural variations of depressive disorders is evident in the measurement of depressive experiences. The existing assessments of depressive symptoms may have limited cultural validity, and this may reduce their clinical utility in non-Western populations [53]. The symptoms of major depression that are described by the DSM-IV and measured by clinicians may not be equally culturally sensitive to depressive experience (i.e., may be endorsed differently) in all populations. Therefore, Marsella [28, 48] proposed to measure depression based on five different dimensions: affective, somatic, interpersonal, cognitive, and existential. According to Marsella, all of these components are present in the depression diagnosis. Yet, in Western culture more attention may be placed on affective and existential symptoms (e.g., depressed mood, discouragement, hopelessness), while non-Western populations may be more likely to experience dysfunction through somatic symptoms (e.g., loss of appetite, sleep problems). To illustrate this, Marsella et al. [54] used factor analysis to explore the expression of depressive symptoms among Japanese, Chinese, and European Americans. The authors found different depressive symptom profiles among the three groups: the Chinese Americans were more likely to emphasize somatic complaints (e.g., headaches, insomnia, and indigestion), the Japanese Americans experienced more interpersonal problems (e.g., afraid to meet new people, does not feel like socializing, and feels ashamed), and the European Americans reported more affective and existential symptoms (e.g., loss of interest in life, hopelessness, depressed mood, suicidality, and memory problems). In addition, the authors found that Chinese and Japanese participants differed from European American participants by reporting poor appetite more often, while European American participants endorsed the urge to eat more frequently than the participants of Asian descent. The findings of this study suggested that various ethnic/racial groups may experience depression in different ways. Therefore, embracing a multidimensional framework to examine the different symptoms of depression and their endorsement and relevance to individuals from various ethnic and racial groups will be an important next step. Learning more about the experience and manifestation of depression across ethnic and racial groups will help us assess, diagnose, and treat depression more effectively.

### 6.3. Intersectionality

As we advance our understanding of the complexity of depression among Asian Americans, we need to consider the effect of other factors, such as gender, acculturation, and language that may influence the manifestation, assessment, diagnosis, and treatment of depression. In particular, the study of intersectionality deals with the “analytic approaches that simultaneously consider the meaning and consequences of multiple categories of identity, difference and disadvantage” [55, page 170].

A few studies concentrated on Asian American women's experience of depression. In a qualitative study of symptom manifestation among Korean immigrant women in the US, Bernstein et al. [56, page 393] found that report of depressive symptoms was complex and in all domains of the person's existence. Some of the topics discussed were “emotional entrapment, shame and failure as women, disappointment at not being able to live a normal life, and emotional restraint”. Women were observed to express emotions more often somatically, bodily, and metaphorically than verbally (e.g., reports of aches and pains and weakness). In addition, women described their experience using the term “suffering” rather than “depression.” The authors explained these patterns with an emphasis on society (collectivism) in Korean culture, where the expression of negative affect may be socially unacceptable. 

Another study examined the relationship among cultural group, depressive symptoms, and somatic symptoms among Japanese and Korean women [57]. The authors found a significant positive correlation between somatic symptoms and high depression scores on the Beck Depression Inventory (BDI) for both cultural groups. The most common endorsed somatic symptoms for both Japanese and Korean women with high BDI scores were abdominal upset, weakness, dizziness, aches and pains, and palpitations. The results of this study suggest that Asian women often tend to express depression in somatic symptoms. The two studies described here concentrated on the depressive experiences of Asian women, but little is known about depression among Asian and Asian American men. Therefore, it is important to investigate more thoroughly depression among Asian American men and women.

Other important factors that may be associated with the expression of depressive symptoms among Asian Americans are acculturation and language. For instance, Chung et al. [20] found that in a primary care setting, 41.3% of Asian patients had depressive symptoms, but physicians identified only 23.6% of them as psychiatrically distressed. The authors concluded that it may be difficult for primary care physicians to recognize depressive symptoms and to give an accurate diagnosis to patients who have low acculturation levels and/or are of Asian ancestry [20]. Similarly, acculturative stress has been positively related to higher rates of depressive symptoms among six groups of Asian immigrant elders (Chinese, Korean, Indian, Filipino, Vietnamese, and Japanese; [58]). In addition, English language proficiency plays an important role in communicating one's symptoms and is an integral part of acculturation [59]. Kim et al. [60] examined the relationship of English proficiency and depressive symptoms in a sample of Chinese American adolescents. The authors found that self-reported low English proficiency in middle school was related to later reporting of accented English in high school, which, in turn, related to their perception of being labeled as perpetual foreigners. Both boys and girls who internalized the perpetual foreigner stereotype experienced more discrimination and reported more depressive symptoms than the adolescents who did not identify as perpetual foreigners. While this study provided insight into the relationship between English proficiency and depression, the role that English proficiency might play in the expression of depressive symptoms among Asian Americans needs to be examined.

In conclusion, it is important to note that the findings reviewed in this paper are based mostly on Asian and European samples from North America. Therefore, their generalizability is limited to this geographical area and their international applicability needs to be examined with caution. The recognition and further exploration of the existing heterogeneity among Asian Americans, the multidimensionality of depression, and the intersectionality of other important variables, such as gender, acculturation, and language seem to be logical next steps for the advancement of our understanding of depression among Asian Americans. Continuing research on separate ethnic Asian groups as opposed to grouping all Asian Americans together may allow us to note similarities and differences in depression between ethnic groups. At the same time, examining depression as a multidimensional construct that consists of various symptoms, as opposed to concentrating on the affective aspect of depression may improve our diagnosis and treatment of depression among Asian Americans. Lastly, considering other variables that play an important role in a person's life and may affect their perceptions of the environment and others, such as gender ability to acculturate and to speak English may prove to be crucial in the assessment, diagnosis, and treatment of depression among Asian Americans.
